# 8-Hy­droxy-2-methyl­quinolinium tetra­chlorido(quinolin-2-olato-κ^2^ *N*,*O*)stannate(IV) methanol disolvate

**DOI:** 10.1107/S1600536811001954

**Published:** 2011-01-22

**Authors:** Ezzatollah Najafi, Mostafa M. Amini, Seik Weng Ng

**Affiliations:** a Department of Chemistry, General Campus, Shahid Beheshti University, Tehran 1983963113, Iran; b Department of Chemistry, University of Malaya, 50603 Kuala Lumpur, Malaysia

## Abstract

In the reaction of 8-hy­droxy­quinoline, 2-methyl-8-hy­droxy­quinoline and stannic chloride, the 2-methyl-8-hy­droxy­quinoline is protonated, yielding the disolvated title salt, (C_10_H_10_NO)[SnCl_4_(C_9_H_6_NO)]·2CH_3_OH. The Sn^IV^ atom in the anion is *N*,*O*-chelated by the hy­droxy­quinolinate in a *cis*-SnNOCl_4_ octa­hedral geometry. In the crystal, the cation, anion and solvent mol­ecules are linked by N—H⋯O, O—H⋯O and O—H⋯Cl hydrogen bonds, generating a three-dimensional network.

## Related literature

For related tin–oxinate structures, see: Archer *et al.* (1987 ▶); Faza­eli *et al.* (2009 ▶); Lo & Ng (2009 ▶).
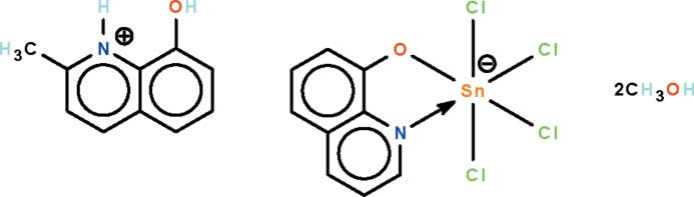


## Experimental

### 

#### Crystal data

(C_10_H_10_NO)[SnCl_4_(C_9_H_6_NO)]·2CH_4_O
*M* *_r_* = 628.91
Triclinic, 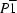

*a* = 7.9395 (3) Å
*b* = 9.9721 (4) Å
*c* = 16.0531 (8) Å
α = 75.056 (4)°
β = 82.529 (4)°
γ = 88.529 (3)°
*V* = 1217.53 (9) Å^3^
*Z* = 2
Mo *K*α radiation
μ = 1.52 mm^−1^
*T* = 100 K
0.30 × 0.25 × 0.20 mm

#### Data collection

Agilent SuperNova Dual diffractometer with an Atlas detector
Absorption correction: multi-scan (*CrysAlis PRO*; Agilent Technologies, 2010 ▶) *T*_min_ = 0.659, *T*_max_ = 0.751
8825 measured reflections
5371 independent reflections
4258 reflections with *I* > 2σ(*I*)
*R*_int_ = 0.040

#### Refinement

*R*[*F* ^2^ > 2σ(*F* ^2^)] = 0.049
*wR*(*F* ^2^) = 0.130
*S* = 1.05
5371 reflections
295 parameters
H-atom parameters constrained
Δρ_max_ = 1.76 e Å^−3^
Δρ_min_ = −1.85 e Å^−3^

### 

Data collection: *CrysAlis PRO* (Agilent Technologies, 2010 ▶); cell refinement: *CrysAlis PRO*; data reduction: *CrysAlis PRO*; program(s) used to solve structure: *SHELXS97* (Sheldrick, 2008 ▶); program(s) used to refine structure: *SHELXL97* (Sheldrick, 2008 ▶); molecular graphics: *X-SEED* (Barbour, 2001 ▶); software used to prepare material for publication: *publCIF* (Westrip, 2010 ▶).

## Supplementary Material

Crystal structure: contains datablocks global, I. DOI: 10.1107/S1600536811001954/si2328sup1.cif

Structure factors: contains datablocks I. DOI: 10.1107/S1600536811001954/si2328Isup2.hkl

Additional supplementary materials:  crystallographic information; 3D view; checkCIF report

## Figures and Tables

**Table 1:** Hydrogen-bond geometry (Å, °)

*D*—H⋯*A*	*D*—H	H⋯*A*	*D*⋯*A*	*D*—H⋯*A*
O2—H2⋯O3	0.84	1.76	2.595 (4)	172
O3—H3⋯O1	0.84	1.91	2.736 (4)	168
O4—H4⋯Cl1^i^	0.84	2.53	3.258 (3)	146
N2—H2n⋯O4	0.88	1.91	2.764 (5)	162

Symmetry code: (i) 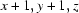.

**Fig. 1.**
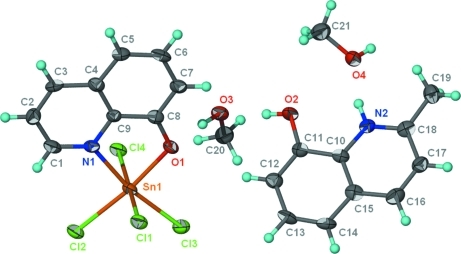
Thermal ellipsoid plot (Barbour, 2001) of [C10H10NO]+ [SnCl4(C9H6NO)]–. 2CH3OH at the 70% probability level; hydrogen atoms are drawn as spheres of arbitrary radius.

## supplementary crystallographic information

### Comment

Only symmetrical dichlorotin bis(oxinates) have been reported; these include the 8-hydroxyquinoline, 2-methyl-8-hydroxyquinoline and 5,7-dichloro-8-hydroxyquinoline derivatives (Archer *et al.*, 1987; Fazaeli *et al.*, 2009; Lo & Ng, 2009). In the reaction of 8-hydroxyquinoline, 2-methyl-8-hydroxyquinoline and stannic chloride, the 2-methyl-8-hydroxyquinoline is protonated to yield the disolvated salt, [SnCl_4_(C_9_H_6_NO_2_)]^-.^2CH_3_OH (Scheme I, Fig. 1). The tin atom in the anion is *N*,*O*-chelated by the hydroxyquinolinate and it exists in a *cis*-SnNOCl_4_ octahedral geometry. The cation, anion and solvent molecules are linked by N–H···O and O–H···O hydrogen bonds to generate a three-dimensional network (Table 1).

### Experimental

Stannic chloride pentahydrate (0.35 g, 1 mmol), 8-hydroxyquinoline (0.15 g, 1 mmol) and 2-methyl-8-hydroxyquinoline (0.16 g, 1 mmol) were loaded into a convection tube; the tube was filled with dry methanol and kept at 333 K. Yellow crystals were collected from the side arm after several days.

### Refinement

Carbon-bound H-atoms were placed in calculated positions [C—H 0.95 to 0.98 Å, *U*_iso_(H) 1.2 to 1.5*U*_eq_(C)] and were included in the refinement in the riding model approximation.

The amino and hydroxy H-atoms were similarly placed (N–H 0.88±0.01, O–H 0.84±0.01 Å) and their temperature factors were also tied. The final difference Fourier map had a peak and a hole in the vicinity of Sn1.

### Crystal data

**Table:**

(C_10_H_10_NO)[SnCl_4_(C_9_H_6_NO)]·2CH_4_O	*Z* = 2
*M**_r_* = 628.91	*F*(000) = 628
Triclinic, *P*1	*D*_x_ = 1.715 Mg m^−^^3^
Hall symbol: -P 1	Mo *K*α radiation, λ = 0.71073 Å
*a* = 7.9395 (3) Å	Cell parameters from 4114 reflections
*b* = 9.9721 (4) Å	θ = 2.6–29.3°
*c* = 16.0531 (8) Å	µ = 1.52 mm^−^^1^
α = 75.056 (4)°	*T* = 100 K
β = 82.529 (4)°	Prism, yellow
γ = 88.529 (3)°	0.30 × 0.25 × 0.20 mm
*V* = 1217.53 (9) Å^3^	

### Data collection

**Table:**

Agilent SuperNova Dual diffractometer with an Atlas detector	5371 independent reflections
Radiation source: SuperNova (Mo) X-ray Source	4258 reflections with *I* > 2σ(*I*)
Mirror	*R*_int_ = 0.040
Detector resolution: 10.4041 pixels mm^-1^	θ_max_ = 27.5°, θ_min_ = 2.6°
ω scans	*h* = −8→10
Absorption correction: multi-scan (*CrysAlis PRO*; Agilent Technologies, 2010)	*k* = −11→12
*T*_min_ = 0.659, *T*_max_ = 0.751	*l* = −16→20
8825 measured reflections	

### Refinement

**Table:**

Refinement on *F*^2^	Primary atom site location: structure-invariant direct methods
Least-squares matrix: full	Secondary atom site location: difference Fourier map
*R*[*F*^2^ > 2σ(*F*^2^)] = 0.049	Hydrogen site location: inferred from neighbouring sites
*wR*(*F*^2^) = 0.130	H-atom parameters constrained
*S* = 1.05	*w* = 1/[σ^2^(*F*_o_^2^) + (0.0633*P*)^2^] where *P* = (*F*_o_^2^ + 2*F*_c_^2^)/3
5371 reflections	(Δ/σ)_max_ = 0.001
295 parameters	Δρ_max_ = 1.76 e Å^−^^3^
0 restraints	Δρ_min_ = −1.85 e Å^−^^3^

### Fractional atomic coordinates and isotropic or equivalent isotropic displacement parameters (Å^2^)

**Table:**

	*x*	*y*	*z*	*U*_iso_*/*U*_eq_	
Sn1	0.51336 (4)	0.56573 (3)	0.20809 (2)	0.01909 (12)	
Cl1	0.24880 (15)	0.54380 (11)	0.30721 (8)	0.0232 (3)	
Cl2	0.47350 (15)	0.33873 (11)	0.18991 (8)	0.0242 (3)	
Cl3	0.67740 (16)	0.50347 (12)	0.32577 (8)	0.0262 (3)	
Cl4	0.75884 (15)	0.60727 (11)	0.09839 (8)	0.0227 (3)	
O1	0.5234 (4)	0.7725 (3)	0.2102 (2)	0.0203 (7)	
O2	0.8013 (5)	1.0336 (3)	0.3161 (2)	0.0280 (8)	
H2	0.8017	0.9885	0.2786	0.042*	
O3	0.8327 (4)	0.8959 (3)	0.1982 (2)	0.0253 (8)	
H3	0.7453	0.8506	0.1976	0.038*	
O4	1.0402 (4)	1.2688 (3)	0.3035 (2)	0.0262 (8)	
H4	1.1279	1.3129	0.3052	0.039*	
N1	0.3617 (5)	0.6649 (4)	0.1035 (2)	0.0191 (8)	
N2	0.8391 (5)	1.1312 (4)	0.4535 (3)	0.0216 (9)	
H2N	0.8831	1.1741	0.4004	0.032*	
C1	0.2875 (6)	0.6087 (5)	0.0509 (3)	0.0235 (10)	
H1	0.2968	0.5117	0.0565	0.028*	
C2	0.1958 (6)	0.6896 (5)	−0.0127 (3)	0.0256 (11)	
H2A	0.1428	0.6470	−0.0492	0.031*	
C3	0.1824 (6)	0.8287 (5)	−0.0226 (3)	0.0215 (10)	
H3A	0.1215	0.8834	−0.0663	0.026*	
C4	0.2598 (6)	0.8921 (5)	0.0328 (3)	0.0211 (10)	
C5	0.2544 (6)	1.0356 (5)	0.0277 (3)	0.0219 (10)	
H5	0.1940	1.0964	−0.0139	0.026*	
C6	0.3369 (6)	1.0866 (5)	0.0830 (3)	0.0247 (11)	
H6	0.3334	1.1834	0.0788	0.030*	
C7	0.4256 (6)	1.0005 (5)	0.1451 (3)	0.0228 (10)	
H7	0.4803	1.0393	0.1828	0.027*	
C8	0.4353 (6)	0.8596 (5)	0.1527 (3)	0.0197 (10)	
C9	0.3486 (6)	0.8058 (4)	0.0956 (3)	0.0171 (9)	
C10	0.7577 (6)	1.0063 (5)	0.4660 (3)	0.0208 (10)	
C11	0.7396 (6)	0.9540 (5)	0.3939 (3)	0.0220 (10)	
C12	0.6582 (6)	0.8283 (5)	0.4091 (3)	0.0238 (11)	
H12	0.6452	0.7911	0.3614	0.029*	
C13	0.5940 (6)	0.7539 (5)	0.4932 (3)	0.0254 (11)	
H13	0.5399	0.6668	0.5013	0.030*	
C14	0.6071 (6)	0.8030 (5)	0.5637 (3)	0.0251 (11)	
H14	0.5609	0.7521	0.6203	0.030*	
C15	0.6919 (6)	0.9330 (5)	0.5509 (3)	0.0226 (10)	
C16	0.7117 (7)	0.9934 (5)	0.6198 (3)	0.0283 (11)	
H16	0.6721	0.9455	0.6781	0.034*	
C17	0.7881 (6)	1.1211 (5)	0.6025 (3)	0.0247 (11)	
H17	0.7957	1.1629	0.6488	0.030*	
C18	0.8548 (6)	1.1910 (5)	0.5179 (3)	0.0224 (10)	
C19	0.9411 (6)	1.3281 (5)	0.4955 (4)	0.0278 (11)	
H19A	0.9106	1.3840	0.4399	0.042*	
H19B	1.0644	1.3150	0.4908	0.042*	
H19C	0.9054	1.3758	0.5410	0.042*	
C20	0.9683 (6)	0.8028 (5)	0.2200 (4)	0.0289 (12)	
H20A	1.0752	0.8552	0.2092	0.043*	
H20B	0.9763	0.7361	0.1843	0.043*	
H20C	0.9470	0.7529	0.2816	0.043*	
C21	0.9985 (7)	1.3030 (6)	0.2168 (3)	0.0319 (12)	
H21A	0.8821	1.2724	0.2171	0.048*	
H21B	1.0773	1.2565	0.1812	0.048*	
H21C	1.0075	1.4037	0.1924	0.048*	

### Atomic displacement parameters (Å^2^)

**Table:**

	*U*^11^	*U*^22^	*U*^33^	*U*^12^	*U*^13^	*U*^23^
Sn1	0.0224 (2)	0.01323 (17)	0.0203 (2)	−0.00323 (12)	−0.00130 (14)	−0.00209 (13)
Cl1	0.0248 (6)	0.0189 (5)	0.0238 (6)	−0.0031 (4)	0.0030 (5)	−0.0041 (5)
Cl2	0.0291 (7)	0.0147 (5)	0.0277 (7)	−0.0021 (4)	−0.0006 (5)	−0.0045 (5)
Cl3	0.0291 (7)	0.0225 (6)	0.0245 (7)	−0.0029 (5)	−0.0066 (5)	0.0002 (5)
Cl4	0.0249 (6)	0.0167 (5)	0.0237 (6)	−0.0025 (4)	0.0021 (5)	−0.0028 (5)
O1	0.0243 (18)	0.0132 (15)	0.0230 (19)	−0.0003 (13)	−0.0024 (15)	−0.0039 (14)
O2	0.041 (2)	0.0218 (17)	0.0186 (19)	−0.0130 (15)	0.0025 (16)	−0.0020 (14)
O3	0.0248 (19)	0.0234 (17)	0.025 (2)	−0.0081 (14)	−0.0014 (15)	−0.0005 (15)
O4	0.030 (2)	0.0241 (18)	0.024 (2)	−0.0085 (14)	−0.0026 (15)	−0.0041 (15)
N1	0.018 (2)	0.0176 (19)	0.019 (2)	−0.0039 (15)	0.0008 (16)	−0.0001 (16)
N2	0.024 (2)	0.021 (2)	0.018 (2)	−0.0018 (16)	−0.0012 (17)	−0.0019 (17)
C1	0.027 (3)	0.015 (2)	0.025 (3)	−0.0030 (19)	0.004 (2)	−0.003 (2)
C2	0.029 (3)	0.029 (3)	0.019 (3)	−0.005 (2)	−0.002 (2)	−0.007 (2)
C3	0.022 (3)	0.020 (2)	0.018 (3)	0.0015 (18)	−0.002 (2)	0.0032 (19)
C4	0.022 (3)	0.019 (2)	0.020 (3)	−0.0034 (18)	0.006 (2)	−0.005 (2)
C5	0.022 (3)	0.019 (2)	0.021 (3)	0.0026 (19)	−0.002 (2)	0.000 (2)
C6	0.026 (3)	0.014 (2)	0.030 (3)	−0.0032 (19)	0.007 (2)	−0.001 (2)
C7	0.024 (3)	0.021 (2)	0.019 (3)	−0.0067 (19)	0.005 (2)	−0.002 (2)
C8	0.016 (2)	0.019 (2)	0.021 (3)	−0.0012 (18)	0.0017 (19)	−0.0030 (19)
C9	0.018 (2)	0.017 (2)	0.014 (2)	−0.0023 (17)	0.0039 (18)	−0.0025 (18)
C10	0.019 (3)	0.017 (2)	0.023 (3)	0.0003 (18)	0.000 (2)	−0.002 (2)
C11	0.023 (3)	0.020 (2)	0.022 (3)	−0.0006 (19)	−0.003 (2)	−0.003 (2)
C12	0.023 (3)	0.023 (2)	0.024 (3)	−0.0014 (19)	0.001 (2)	−0.005 (2)
C13	0.023 (3)	0.016 (2)	0.034 (3)	−0.0063 (19)	0.004 (2)	−0.002 (2)
C14	0.030 (3)	0.016 (2)	0.024 (3)	0.0005 (19)	0.002 (2)	0.003 (2)
C15	0.016 (2)	0.022 (2)	0.028 (3)	0.0051 (19)	−0.001 (2)	−0.005 (2)
C16	0.031 (3)	0.031 (3)	0.020 (3)	0.002 (2)	0.001 (2)	−0.003 (2)
C17	0.026 (3)	0.028 (3)	0.019 (3)	−0.001 (2)	−0.002 (2)	−0.005 (2)
C18	0.014 (2)	0.028 (3)	0.024 (3)	0.0036 (19)	−0.002 (2)	−0.004 (2)
C19	0.023 (3)	0.030 (3)	0.032 (3)	−0.002 (2)	−0.006 (2)	−0.011 (2)
C20	0.025 (3)	0.031 (3)	0.029 (3)	−0.002 (2)	−0.004 (2)	−0.005 (2)
C21	0.032 (3)	0.034 (3)	0.027 (3)	−0.004 (2)	−0.004 (2)	−0.002 (2)

### Geometric parameters (Å, °)

**Table:**

Sn1—O1	2.075 (3)	C6—H6	0.9500
Sn1—N1	2.204 (4)	C7—C8	1.379 (6)
Sn1—Cl3	2.3758 (12)	C7—H7	0.9500
Sn1—Cl2	2.3898 (11)	C8—C9	1.428 (6)
Sn1—Cl4	2.4174 (12)	C10—C15	1.409 (7)
Sn1—Cl1	2.4427 (12)	C10—C11	1.412 (6)
O1—C8	1.350 (5)	C11—C12	1.376 (6)
O2—C11	1.333 (6)	C12—C13	1.399 (7)
O2—H2	0.8400	C12—H12	0.9500
O3—C20	1.423 (6)	C13—C14	1.361 (7)
O3—H3	0.8400	C13—H13	0.9500
O4—C21	1.426 (6)	C14—C15	1.431 (7)
O4—H4	0.8400	C14—H14	0.9500
N1—C1	1.328 (6)	C15—C16	1.416 (6)
N1—C9	1.381 (5)	C16—C17	1.371 (7)
N2—C18	1.341 (6)	C16—H16	0.9500
N2—C10	1.373 (6)	C17—C18	1.397 (7)
N2—H2N	0.8800	C17—H17	0.9500
C1—C2	1.400 (7)	C18—C19	1.483 (7)
C1—H1	0.9500	C19—H19A	0.9800
C2—C3	1.358 (6)	C19—H19B	0.9800
C2—H2A	0.9500	C19—H19C	0.9800
C3—C4	1.422 (6)	C20—H20A	0.9800
C3—H3A	0.9500	C20—H20B	0.9800
C4—C9	1.399 (7)	C20—H20C	0.9800
C4—C5	1.411 (6)	C21—H21A	0.9800
C5—C6	1.370 (6)	C21—H21B	0.9800
C5—H5	0.9500	C21—H21C	0.9800
C6—C7	1.393 (7)		
			
O1—Sn1—N1	78.45 (12)	N1—C9—C4	121.7 (4)
O1—Sn1—Cl3	90.22 (9)	N1—C9—C8	116.6 (4)
N1—Sn1—Cl3	168.60 (9)	C4—C9—C8	121.7 (4)
O1—Sn1—Cl2	171.51 (9)	N2—C10—C15	119.3 (4)
N1—Sn1—Cl2	93.09 (10)	N2—C10—C11	119.7 (4)
Cl3—Sn1—Cl2	98.22 (4)	C15—C10—C11	121.0 (4)
O1—Sn1—Cl4	88.73 (9)	O2—C11—C12	125.8 (4)
N1—Sn1—Cl4	87.00 (10)	O2—C11—C10	116.2 (4)
Cl3—Sn1—Cl4	93.98 (4)	C12—C11—C10	118.0 (5)
Cl2—Sn1—Cl4	91.61 (4)	C11—C12—C13	121.5 (4)
O1—Sn1—Cl1	88.01 (9)	C11—C12—H12	119.3
N1—Sn1—Cl1	86.76 (10)	C13—C12—H12	119.3
Cl3—Sn1—Cl1	91.73 (4)	C14—C13—C12	121.7 (4)
Cl2—Sn1—Cl1	90.78 (4)	C14—C13—H13	119.1
Cl4—Sn1—Cl1	173.44 (4)	C12—C13—H13	119.1
C8—O1—Sn1	114.7 (2)	C13—C14—C15	118.7 (5)
C11—O2—H2	109.5	C13—C14—H14	120.7
C20—O3—H3	109.5	C15—C14—H14	120.7
C21—O4—H4	109.5	C10—C15—C16	117.8 (4)
C1—N1—C9	119.6 (4)	C10—C15—C14	119.1 (4)
C1—N1—Sn1	129.6 (3)	C16—C15—C14	123.1 (5)
C9—N1—Sn1	110.8 (3)	C17—C16—C15	120.0 (5)
C18—N2—C10	123.4 (4)	C17—C16—H16	120.0
C18—N2—H2N	118.3	C15—C16—H16	120.0
C10—N2—H2N	118.3	C16—C17—C18	121.2 (4)
N1—C1—C2	121.3 (4)	C16—C17—H17	119.4
N1—C1—H1	119.3	C18—C17—H17	119.4
C2—C1—H1	119.3	N2—C18—C17	118.2 (4)
C3—C2—C1	120.3 (4)	N2—C18—C19	118.2 (4)
C3—C2—H2A	119.8	C17—C18—C19	123.6 (4)
C1—C2—H2A	119.8	C18—C19—H19A	109.5
C2—C3—C4	119.8 (4)	C18—C19—H19B	109.5
C2—C3—H3A	120.1	H19A—C19—H19B	109.5
C4—C3—H3A	120.1	C18—C19—H19C	109.5
C9—C4—C5	118.5 (4)	H19A—C19—H19C	109.5
C9—C4—C3	117.2 (4)	H19B—C19—H19C	109.5
C5—C4—C3	124.3 (4)	O3—C20—H20A	109.5
C6—C5—C4	119.6 (4)	O3—C20—H20B	109.5
C6—C5—H5	120.2	H20A—C20—H20B	109.5
C4—C5—H5	120.2	O3—C20—H20C	109.5
C5—C6—C7	121.8 (4)	H20A—C20—H20C	109.5
C5—C6—H6	119.1	H20B—C20—H20C	109.5
C7—C6—H6	119.1	O4—C21—H21A	109.5
C8—C7—C6	120.9 (4)	O4—C21—H21B	109.5
C8—C7—H7	119.5	H21A—C21—H21B	109.5
C6—C7—H7	119.5	O4—C21—H21C	109.5
O1—C8—C7	123.1 (4)	H21A—C21—H21C	109.5
O1—C8—C9	119.4 (4)	H21B—C21—H21C	109.5
C7—C8—C9	117.5 (4)		
			
N1—Sn1—O1—C8	1.6 (3)	C5—C4—C9—N1	−178.9 (4)
Cl3—Sn1—O1—C8	−177.2 (3)	C3—C4—C9—N1	0.3 (7)
Cl4—Sn1—O1—C8	88.8 (3)	C5—C4—C9—C8	−1.1 (7)
Cl1—Sn1—O1—C8	−85.5 (3)	C3—C4—C9—C8	178.1 (4)
O1—Sn1—N1—C1	178.1 (4)	O1—C8—C9—N1	−0.1 (6)
Cl3—Sn1—N1—C1	−175.9 (4)	C7—C8—C9—N1	179.2 (4)
Cl2—Sn1—N1—C1	−2.6 (4)	O1—C8—C9—C4	−178.0 (4)
Cl4—Sn1—N1—C1	88.8 (4)	C7—C8—C9—C4	1.3 (7)
Cl1—Sn1—N1—C1	−93.2 (4)	C18—N2—C10—C15	−2.4 (7)
O1—Sn1—N1—C9	−1.6 (3)	C18—N2—C10—C11	176.9 (4)
Cl3—Sn1—N1—C9	4.4 (7)	N2—C10—C11—O2	−1.5 (7)
Cl2—Sn1—N1—C9	177.6 (3)	C15—C10—C11—O2	177.8 (4)
Cl4—Sn1—N1—C9	−90.9 (3)	N2—C10—C11—C12	179.7 (4)
Cl1—Sn1—N1—C9	87.0 (3)	C15—C10—C11—C12	−1.0 (7)
C9—N1—C1—C2	0.0 (7)	O2—C11—C12—C13	−178.4 (5)
Sn1—N1—C1—C2	−179.7 (3)	C10—C11—C12—C13	0.3 (7)
N1—C1—C2—C3	0.7 (8)	C11—C12—C13—C14	0.8 (8)
C1—C2—C3—C4	−0.9 (8)	C12—C13—C14—C15	−1.2 (7)
C2—C3—C4—C9	0.4 (7)	N2—C10—C15—C16	0.7 (7)
C2—C3—C4—C5	179.5 (5)	C11—C10—C15—C16	−178.7 (4)
C9—C4—C5—C6	0.7 (7)	N2—C10—C15—C14	179.9 (4)
C3—C4—C5—C6	−178.5 (5)	C11—C10—C15—C14	0.6 (7)
C4—C5—C6—C7	−0.5 (8)	C13—C14—C15—C10	0.5 (7)
C5—C6—C7—C8	0.7 (8)	C13—C14—C15—C16	179.7 (5)
Sn1—O1—C8—C7	179.3 (4)	C10—C15—C16—C17	2.0 (7)
Sn1—O1—C8—C9	−1.4 (5)	C14—C15—C16—C17	−177.2 (5)
C6—C7—C8—O1	178.2 (4)	C15—C16—C17—C18	−3.1 (7)
C6—C7—C8—C9	−1.1 (7)	C10—N2—C18—C17	1.4 (7)
C1—N1—C9—C4	−0.5 (7)	C10—N2—C18—C19	−178.2 (4)
Sn1—N1—C9—C4	179.3 (4)	C16—C17—C18—N2	1.4 (7)
C1—N1—C9—C8	−178.4 (4)	C16—C17—C18—C19	−179.0 (4)
Sn1—N1—C9—C8	1.4 (5)		

### Hydrogen-bond geometry (Å, °)

**Table:**

*D*—H···*A*	*D*—H	H···*A*	*D*···*A*	*D*—H···*A*
O2—H2···O3	0.84	1.76	2.595 (4)	172
O3—H3···O1	0.84	1.91	2.736 (4)	168
O4—H4···Cl1^i^	0.84	2.53	3.258 (3)	146
N2—H2n···O4	0.88	1.91	2.764 (5)	162

Symmetry codes: (i) *x*+1, *y*+1, *z*.
